# Efficacy and tolerability of lacosamide as adjunctive therapy in patients with focal-onset seizures: an observational, prospective study

**DOI:** 10.1007/s13760-023-02236-8

**Published:** 2023-04-01

**Authors:** Yang Jin, Ranran Zhang, Jing Jiang, Xuewu Liu

**Affiliations:** 1 Department of Neurology, Qilu Hospital of Shandong University, Cheeloo College of Medicine, 107 Jinan Culture Road, Jinan, 250012 Shandong China; 2 Institute of Epilepsy, Shandong University, Jinan, China

**Keywords:** Epilepsy, Anti-seizure medication, Focal seizures, Lacosamide, Seizure freedom

## Abstract

**Purpose:**

To evaluate the efficacy and tolerability of adjunctive lacosamide (LCM) in patients with focal-onset seizures, with or without combined secondarily generalized seizures.

**Methods:**

106 patients aged ≥ 16 years were recruited consecutively in this single-center prospective observational study. All patients received LCM as an add-on treatment on the basis of clinical judgement. Seizure frequency, adverse events (AEs) and retention rates were obtained at 3 and 6 months after LCM introduction.

**Result:**

The overall response rates were 53.3 and 70.4% after 3 and 6 months, respectively, and the freedom of seizures at the same points was reached at 19 and 26.5%. The retention rates were 99.1% at the 3-month follow-up and 93.3% at the 6-month follow-up. The overall incidence of adverse events was 35.8%. The leading AEs were dizziness (16.98%) and sedation (6.6%).

**Conclusions:**

Our study confirmed the efficacy and tolerability of adjunctive LCM in Chinese patients in real-life conditions. Based on our treatment experience, a universal maintenance dose of LCM would be needed in Chinese patients.

## Introduction

Epilepsy is one of the most common chronic diseases of the central nervous system, with an estimated prevalence of 0.5–1% [1]. In China, The prevalence of epilepsy is about 7.0 per 1000 [2]. Anti-seizure medication (ASM) therapy is the main treatment for epilepsy, but about 30% of patients still do not successfully respond to ASMs [3]. The International League Against Epilepsy (ILAE) defines drug-resistant epilepsy (DRE) as " failure of adequate trials of two tolerated and appropriately chosen and used AED schedules (whether as monotherapies or in combination) to achieve sustained seizure freedom [4]. Therefore, there is an urgent need to develop and apply newer ASMs with novel mechanisms of action.

Lacosamide, one of the latest anti-epileptic drugs, is a functionalized amino acid with the chemical name (R) -2-acetyl-n-benzyl-3-methoxyacrylamide, which was widely used in adult and childhood epilepsy [5–10]. They function by selectively enhancing the slow inactivation of voltage-gated sodium channels without affecting fast inactivation; in this way, they reduce the excitability of pathological neurons without affecting physiological neuronal function [11]. Meanwhile, it appears to bind to collapsin response mediator protein 2 (CRMP-2) involved in neurotrophic signaling. This is hypothesized to produce a neuroprotective effect preventing the formation of abnormal neuronal connections in the brain [12]. In addition, Ruffolo G et al. [13] have recently found that lacosamide also acts on the GABA receptor; this mechanism is related to its ability to prevent the evolution of status epilepticus.

Therefore, we conducted this prospective study to evaluate the efficacy and tolerability of LCM as an add-on therapy in patients with focal-onset seizures, with or without secondarily generalized seizures.

## Methods

### Subjects

Patients with focal-onset seizures (with or without secondarily generalized seizures) who visited Qilu Hospital of Shandong University from August 2020 to April 2021 were enrolled in this study. The study was performed in accordance with the Declaration of Helsinki. All subjects and their guardians (in the case of children) gave informed consent to participate. The inclusion criteria were: (a) age ≥ 16 years old; (b) diagnosed as focal-onset seizures [14, 15] (with or without secondarily generalized seizures) based on clinical presentations and EEG confirmation, according to International League Against Epilepsy classifications of seizures and epilepsy; (c) the patients have ≥ 1 seizures per month during the 3 months before inclusion; (d) seizures were not controlled with ≥ 1 ASM. The exclusion criteria: (a) psychogenic nonepileptic seizures; (b)status epilepticus; (c)primary generalized tonic–clonic seizures; (d) lactation or pregnancy; (e) alcohol or drug abuse; (f) psychiatric disease and severe diseases of other systems; patients were excluded if they had experienced these symptoms during the 3 months before inclusion.

### Study design

This was a 6 month, single-center, prospective study evaluating the efficacy and safety of LCM. The following clinical data of the patients were collected: age, sex, etiology of epilepsy, age at onset, duration of epilepsy, previous ASMs, concomitant ASMs, seizure frequency (monthly seizure frequency during the 3 months before starting lacosamide treatment), seizure types (focal or combined secondarily generalized seizures). Enzyme-inducing ASMs (EIASMs) included carbamazepine, oxcarbazepine, phenobarbital, phenytoin. All patients enrolled were treated with LCM twice daily at morning time and bedtime, the initial dose was 50 mg each time, the maintenance dose was 100 mg each time after two weeks, all patients were used to the same dosing schedules. And there was no change in concomitant ASMs (neither changes in types of concomitant ASMs nor changes in doses of concomitant ASMs). Patients were followed up at 3 and 6 months after LCM add-on.

### Outcome evaluation

Efficacy was assessed by measuring changes in seizure frequency at 3 and 6 months follow-up compared with baseline. The baseline was 3 months before the addition of LCM and the seizure frequency was based on the patients’ seizure diary. We classified patients into five categories: (1) seizure freedom, (2) reduction of seizure frequency ≥ 50%, (3) reduction of seizure frequency < 50%, (4) no change, (5) worsened (patients with increased seizure frequency). Patients who have achieved a reduction of more than 50% or seizure free were defined as responders (The criteria for patients with combined secondarily generalized seizures was to achieve a reduction in all seizure types).

Tolerability was evaluated by reported adverse events (AEs), LCM discontinuation and clinical laboratory tests (blood routine, blood sedimentation rate, blood glucose, blood lipid, liver and kidney function, blood electrolytes, and urine routine) at each follow-up. AEs were mainly based on directly reported by the patients and through specific questioning about the most known common AEs associated with LCM (no standardized AE questionnaire was used).

Adverse reactions are mainly manifested as dizziness, sedation, fatigue, memory loss, nausea, irritability, vision and gait instability. Those who cannot follow-up timely due to COVID-19 will be follow-up by telephone or WeChat.

### Statistical analysis

All statistical analyses were performed using the statistical software SPSS version 23.0. For between-group comparisons, *t* Tests or the non-parametric Mann–Whitney *U* tests were used to compare continuous variables, while categorical variables were compared using Chi-squared tests or Fisher’s exact test. Retention rate was calculated by counting the number of patients taking lacosamide every 2 weeks using the Kaplan–Meier survival analysis. The threshold of statistical significance was *p* < 0.05.

## Result

### Subjects

We enrolled 111 patients, but five of them did not receive any dose of lacosamide (three patients refused to sign the informed consent, two patients worried about adverse events of LCM), so their data were not included in this study, we ultimately recruited 106 patients (47 females, 59 males). The mean age was 33.1 ± 13.7 years (range 16–71 years). Baseline population and clinical characteristics of the patients are shown in Table 1. The etiology was classified according to the International Anti-epilepsy Consortium, including genetic (*n* = 1), structural (*n* = 27), immunity (*n* = 2), metabolism (*n* = 3), infection (*n* = 14) and unknown etiology (*n* = 59). Among all patients enrolled in the study, 32(30.2%) had focal seizures and 74 (69.8%) had secondarily generalized seizures, and their monthly seizure frequency was 13.4 ± 22.

**Table 1:** Demographical and clinical characteristics of patients

Total (*n*)	106
Age, years (mean ± SD)	33.1 ± 13.7
Sex, Male/Female, *n* (%)	47/59 (44.3/55.7)
Age at epilepsy onset, years (mean ± SD)	26.7 ± 14.5
Epilepsy duration, years (mean ± SD)	7.7 ± 8.2
Etiology	
Structural, *n* (%)	27 (25.47)
Genetic, *n* (%)	1 (0.94)
Immune, *n* (%)	2 (1.89)
Metabolic, *n* (%)	3 (2.83)
Infection, *n* (%)	14 (13.21)
Unknown, *n* (%)	59 (55.66)
Seizure type	
Focal, *n* (%)	32 (30.2)
sGTCS, *n* (%)	74 (69.8)
Monthly seizure frequency(mean ± SD)	13.4 ± 21.9
Previous ASMs (mean ± SD)	2.65 ± 0.72
Concomitant ASMs (mean ± SD)	1.65 ± 0.71
Concomitant EIASMs, *n* (%)	37 (34.9)
Concomitant Non-EIASMs, *n* (%)	69 (65.1)

*sGTCS* secondarily generalized tonic–clonic seizure, *ASMs* Anti-seizure medications, *EIASMs* enzyme inducer AEDs

All patients were taking at least one ASM at the time of initiation of LCM, while the mean number of ASM was 1.65 ± 0.71 and the type of accompanying ASM is shown in Table 2. Valproate acid (VPA) is the most common concomitant ASM (73.58%), followed by levetiracetam (LEV)(33.96%), oxcarbazepine (OXC) (17.92%) and carbamazepine(CBZ)(16.04%). Meanwhile, concomitant ASMs were also classified as EIASMs and non-EIASMs (any other ASMs).

**Table 2:** Anti-seizure medications combined with Lacosamide

Concomitant ASMs	*n* (%)
VPA	78 (73.58)
LEV	36 (33.96)
OXC	19 (17.92)
CBZ	17 (16.04)
LTG	11 (10.37)
TPM	11 (10.37)
ZNS	1 (0.94)

*VPA* Valproic acid, *LEV* levetiracetam, *OXC* oxcarbamazeine, *CBZ* carbamazepine, *LTG* lamotrigine, *TPM* topiramate, *ZNS* zonisamide

### LCM dose and retention rate

The starting dose is 100 mg/d, and then increased to 200 mg/d two weeks later to reach a universally tolerated dose with good seizure control. The usual target dose is 400 mg/d, but the dose adjustment is determined by the physician based on the patients’ clinical response.

The retention rate at 3 months was 99.1%(105/106) because one discontinued due to a worsening in seizure frequency. The retention rate at 6 months was 93.3%(98/105) because two patients were lost to follow-up and five patients discontinued lacosamide. Of these five patients, due to AEs occurrence in two subjects, no change in seizure frequency in four patients and a worsening of seizure frequency in one patient.

### Efficacy

**Fig. 1:**
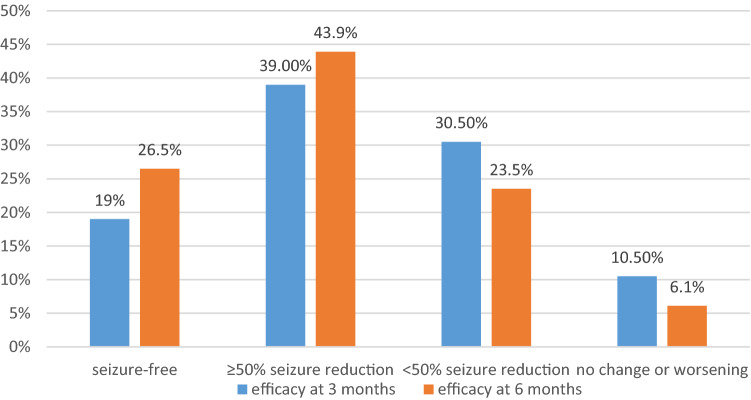
Efficacy of LCM at 3 and 6 months follow-up

At 3 months, 105 patients were available for efficacy evaluations. The overall response rate (≥ 50% seizure reduction) at 3 months was 53.3% (56/105). In detail, seizure freedom was achieved in 19% (20/105), 39.0% (36/105) had a reduction ≥ 50%. 32(30.5%) had a reduction < 50%, 11 (10.5%) showed no change in seizure frequency, and 1(0.9%) out of 105 patients experienced a worsening in seizure frequency (Fig. 1).

At the 6 months follow-up, 98 patients were available for efficacy evaluations. The total response rate at 6 months was 70.4% (69/98), and 26.5% (26/98) became seizure-free, 43.9% (43/98) had a reduction ≥ 50% in seizure frequency. 23 (23.5%) had a reduction < 50% and 6(6.1%) showed no change in seizure frequency. Furthermore, none of the patient experienced a worsening of seizure frequency.

After the last follow-up, patients were divided into responders and non-responders, and comparisons of their demographic and related variables were shown in Table 3. There was no significant difference between responders and non-responders in age, sex, age of onset and so on. We found that patients with shorter epilepsy duration had a better response effect for lacosamide addition, compared with longer epilepsy duration (6.2 ± 5.4 vs 12.2 ± 12.0, *p* = 0.002). And, interestingly, patients with unknown etiology (56/98, 57.1%) and structural (25/98, 25.5%) appeared to respond better, although this result was not statistically significant.

**Table 3:** Comparison of demographics and related variables between responders and non-responders

	Responders	Non-responders	*P*
Age, years (mean ± SD)	33.2 ± 13.6	33.8 ± 13.4	0.727
Sex, Male/Female, *n* (%)	40/29 (40.8/29.6)	14/15 (14.3/15.3)	0.091
Age at epilepsy onset, years (mean ± SD)	26.8 ± 14.7	22.4 ± 12.8	0.336
Epilepsy duration, years (mean ± SD)	6.2 ± 5.4	12.2 ± 12.0	0.002
Etiology			
Structural, *n* (%)	15 (15.3)	10 (10.2)	
Genetic, *n* (%)	0 (0.0)	1 (1.0)	
Immune, *n* (%)	2 (2.0)	0 (0.0)	
Metabolic, *n* (%)	2 (2.0)	0 (0.0)	
Infection, *n* (%)	7 (7.1)	5 (5.1)	
Unknown, *n* (%)	43 (43.9)	13 (13.3)	
Seizure type			
Focal, *n* (%)	22 (22.4)	8 (8.2)	
sGTCS, *n* (%)	46 (48.0)	21 (21.4)	
Monthly seizure frequency(mean ± SD)	14.2 ± 23.0	13.2 ± 21.6	0.986
Previous ASMs	2.0 ± 0.7	2.5 ± 0.8	0.199
Mean number of concomitant ASMs	1.6 ± 0.7	1.9 ± 0.7	0.136
Concomitant EIASM(s)			0.050
Concomitant EIASMs	23 (23.5)	13 (13.3)	
Concomitant Non-EIASMs	46 (46.9)	16 (16.3)	
Concomitant ASM(s)			
VPA	59 (60.2)	21 (21.4)	
LEV	22 (22.4)	10 (10.2)	
OXC	11 (11.2)	6 (6.1)	
CBZ	12 (12.2)	5 (5.1)	
LTG	3 (3.1)	7 (7.1)	
TPM	8 (8.2)	4 (4.1)	
ZNS	1 (1.0)	0 (0.0)	

*sGTCS* secondarily generalized tonic–clonic seizure, *ASMs* Anti-seizure medications, *EIASMs* enzyme inducer ASMs, *VPA* Valproic acid, *LEV* levetiracetam, *OXC* oxcarbamazeine, *CBZ* carbamazepine, *LTG* lamotrigine, *TPM* topiramate, *ZNS* zonisamide

Patients combined with secondarily generalized seizures had a higher response rate (48.0%, 47/98) (*p* = 0.168) and non-responders had higher baseline episodes than responders (*p* = 0.986) but were not statistically significant. In addition, there were no significant differences between responders and non-responders in the number of previous ASMs and concomitant ASMs (*p* = 0.136).

Patients concomitant with non-EIASM (77.4%, 48/62) responded higher than those with EIASM(58.3%,21/36) but not statistically significant(*p* = 0.05), and in concurrent ASM, VPA and LEV combined more frequently in responders than non-responders. In addition, the proportion of responders combining VPA and LCM was highest (36.2%, 25/69), followed by the LEV and LCM combination (13.0%, 9/69).

### Safety

**Fig. 2:**
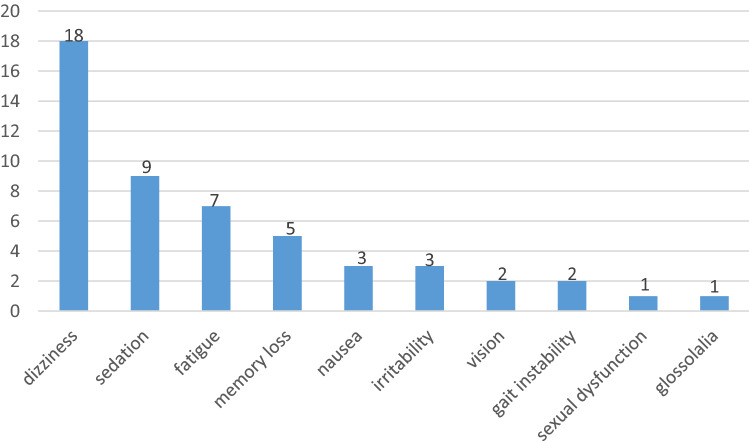
Adverse events associated with LCM treatment

Overall, 38 (35.8%) out of the 106 patients experienced at least one adverse event during treatment with LCM (6-month follow-up), and a total of 51 adverse events were reported (Fig. 2). There were no significant differences in the daily LCM doses between patients experiencing adverse events and those without. Dizziness was the most common adverse reaction (16.98%, 18/106), followed by sedation (8.5%, 9/106) and fatigue (6.6%, 7/106). Memory loss (4.71%, 5/106), nausea (2.83%, 3/106), irritability (2.83%, 3/106), vision (1.87%, 2/106) and gait instability (1.87%, 2/106) were less frequently mentioned. In general, adverse events were mild to moderate and could be tolerated by most patients. Three (2.83%) stopped due to adverse events (some had more than one adverse event). Specifically, one patient withdrew from LCM treatment for dizziness and gait instability, one patient for lethargy, and one for memory loss.

We also observed some changes in laboratory examination, but most were within the normal range and had no clinical significance.

## Discussion

Surgical treatment for epilepsy achieved seizure-free four times than medication [15]. However, a significant proportion of refractory patients are either ineligible for surgery or refuse surgery. In addition, approximately one-third of the patients had a persistent seizure episode after seizure surgery [16, 17]. Thus, medication remains the primary treatment for most patients with focal seizures. In patients with focal epilepsy, combination therapy is a more effective treatment regimen after the first AED failed due to lack of efficacy [18]. A large sample population study showed that combination medications treatments with different mechanisms of action showed greater efficacy compared to the same mechanism [19]. The mechanism of ASM should be considered when selecting the second ASM as a combination treatment. Combination therapy with the old enzyme-induced ASM may reduce the efficacy of other drugs [20]. The use of novel non-enzyme-induced ASM is easier to implement combination treatment [21]. And patients with shorter epilepsy duration had a better response effect for lacosamide addition, compared with longer epilepsy duration, this finding would encourage the early initiation of lacosamide. Since 2010, numerous studies have demonstrated the good clinical efficacy of LCM in patients with epilepsy [22–25], and this observational study evaluated the efficacy and safety of LCM as an add-on therapy for the treatment of patients with focal-onset seizures, with or without secondarily generalized seizures. In our population, we found a responder rate of 53.3% after 3 months and 70.4% after 6 months of LCM administration, with 19% and 26.5% of patients achieving seizure freedom at 3 and 6 months, respectively.

In combination therapy, common combination agents include multitarget ASMS, synaptic vesicle proteins 2A regulators, fast sodium channel blockers, benzodiazepines, etc. Lacosamide has ideal pharmacokinetics and pharmacodynamic properties, which was no need to consider clinical interaction [26]; however, in clinical practice, it has been found to have potential effects with other drugs. Serum concentrations were affected by enzyme inducers and age, and Tountopoulou M et al. [27] reported that lacosamide reduced blood concentrations of sodium valproate acid (VPA) and levetiracetam (LEV). The mechanism of this change is not completely understood. Markoula S et al. [28] reported that carbamazepine and sodium phenytoin may significantly reduce serum lacosamide concentrations through the induction of lysamine metabolism. Simona Lattanzi et al. [29] also reported that there was a statistically significant (*p* = 0.006) higher number of responders to BRV in patients who were receiving SCBs than in those receiving no sodium channel blockers (SCBs), and treatment discontinuation due to AEs was less common in patients treated with BRV and concomitant SCBs than in patients treated with BRV and no SCBs (8.8% vs. 14.6%; *p* = 0.038). The chemical structure of brivaracetam (BRV) is similar to levetiracetam (LEV).

The most common adverse events of LCM involve the nervous and gastrointestinal systems, including headache, vomiting, dizziness, nausea, fatigue, ataxia, abnormal vision abnormalities, dizziness, nystagmus, coordination problems and gait problems, with occasional reported drug rash or arrhythmias [30, 31]. Dose-related adverse events include dizziness, nausea, fatigue and vomiting; most are mild or moderate intensity, adverse reactions can be mitigated or disappeared by reducing the maintenance dose [32]. In this experiment, the adverse effects were mild and only 1 patient increased seizure frequency after the addition of LCM and stopped LCM use due to ataxia. These AEs subsided with dosage reduction and withdrawal without causing serious diseases. Moreover, it is worth mentioning that the occurrence of AEs cannot be attributed to a single addition of a new drug, but may also be caused by different combinations and the total drug load [33]. At each follow-up, we performed clinical laboratory tests on the patient, no patient was found to have abnormalities of liver and kidney function during the medication. Some patients had abnormalities in blood glucose and lipid, none were related to our study and they all were long-term chronic diseases in patients.

There are several limitations to our study. First, this was a single-center observational study that included only a limited number of patients. Therefore, randomized double-blind clinical trials on a larger number of patients are necessary for the future. Second, serum levels of LCM were not measured, we could not precisely assess the influence of enzyme inducers on plasma levels associated with seizure control and AEs. Third, due to some patients having 1 seizure per month during the 3 months before inclusion, in the 6 month follow-up time, if they did not show any epileptic seizures within 6 months, it is listed as seizure-free. The follow-up time is short, there may be some deviation, and a longer follow-up is needed for verification. Finally, as we did not use a standardized questionnaire for assessing AEs, the incidence of adverse events caused by LCM treatment may be inaccurate.

This study confirms the good efficacy and tolerance of LCM addition in the treatment of focal epilepsy. As the third generation of ASMs, LCM currently uses insufficient clinical data in China, which may extend the clinical indication of STP beyond DS. Controlled studies with larger samples are needed to confirm these results, and it is believed that future LCM may be a new option for the effective treatment of patients with focal seizures.

## Conclusion

In conclusion, as a prospective real-life study in Chinese patients, our findings confirmed the good efficacy and tolerability of adjunctive LCM in patients with focal-onset seizures, with or without combined secondarily generalized seizures. The concomitant use of EIASMs did not appear to reduce the efficacy of LCM in seizure control. The combined application of valproic acid, levetiracetam and LCM may provide a better treatment option for patients, but further studies with long-term follow-up are necessary to confirm this result.

## Data Availability

All our data came from the epilepsy patient database of our institute, and all data were used with the informed consent of patients to ensure data availability.
